# Effects of an intravenous lidocaine bolus before tracheal extubation on recovery after breast surgery – Lidocaine at the End (LATE) study: a randomized controlled clinical trial

**DOI:** 10.3325/cmj.2023.64.222

**Published:** 2023-08

**Authors:** Boris Mraovic, Tatjana Šimurina

**Affiliations:** 1 Department of Anesthesiology & Perioperative Medicine, University of Missouri – Columbia, Columbia, Missouri; 2 Department of Health Studies, University of Zadar, Zadar, Croatia; 3 Department of Anesthesiology and ICU, Zadar General Hospital, Zadar, Croatia; 4 Faculty of Medicine, University of Osijek, Osijek, Croatia

## Abstract

**Aim:**

To investigate whether IV lidocaine improves emergence, early recovery, and late recovery after general anesthesia in women who undergo breast surgery.

**Methods:**

Sixty-seven women with American Society of Anesthesiologists physical status I-II, scheduled for breast surgery were randomized to receive an IV lidocaine 1.5 mg/kg bolus (n = 34) or saline placebo (n = 33) before tracheal extubation. Anesthesia was induced with thiopental, vecuronium, and fentanyl, and maintained with sevoflurane ~ 1 MAC and 50% nitrous-oxide in oxygen. No postoperative nausea and vomiting (PONV) prophylaxis was given. Time to extubation, bucking before extubation, and quality of emergence, as well as early and late recovery (coughing post-extubation, sore throat, PONV, and pain scores) within 24 hours postoperatively were evaluated. Diclofenac and meperidine were used for the treatment of pain and metoclopramide for PONV.

**Results:**

The groups did not significantly differ in demographics, intraoperative data, or PONV risk scores. Extubation was ~ 8 minutes in both groups. Patients who received IV lidocaine had significantly smoother recovery, both statistically and clinically; they had better extubation quality scores (1.5 [1-3] vs 3 [1-5], *P* < 0.001), less bucking before extubation (38% vs 91%, *P* < 0.001), less coughing after extubation (at 1 min 18% vs 42%, *P* = 0.026; and at 24 hours 9% vs 27%, *P* = 0.049), and less sore throat (6% vs 48%, *P* < 0.001). Late PONV decreased (3% vs 24%, *P* = 0.013). There were no differences in pain scores and treatment.

**Conclusion:**

In women who underwent breast surgery, IV lidocaine bolus administered just before extubation attenuated bucking, cough and sore throat, and PONV for 24 hours after general anesthesia, without prolonging the emergence.

ISRCTN: 71855856.

Smooth emergence and recovery from anesthesia are an important part of anesthesia practice. Avoiding bucking and coughing on emergence reduces multiple postoperative complications ranging from wound dehiscence and hematomas, especially in plastic and hernia repair surgeries, to an increase in hemodynamic response, which could lead to acute cardiovascular events and increased intraocular and intracranial pressure. One way of reducing bucking and coughing on emergence is IV lidocaine given before endotracheal extubation. A recent meta-analysis of 16 studies (n = 1516) showed that early recovery was improved by administration of 1-1.5 mg/kg of IV lidocaine (1). The meta-analysis included all studies regardless of timing when lidocaine was given, either preoperatively (pre-induction or on induction) or intraoperatively (from the end of surgery, on wound closure, on reversal of neuromuscular blockade up to two minutes before extubation). It showed large reductions in post-extubation cough (risk ratio [RR] 0.64; 95% confidence interval [CI] 0.48-0.86) and in postoperative sore throat at 1 h (RR 0.46; 95% CI 0.32-0.67) but not in late recovery or post-operative nausea and vomiting (PONV) (1).

Since the data about the influence of pre-extubation lidocaine on early and late recovery are sparse, we investigated whether IV lidocaine would improve emergence and prevent complications in early and late recovery after general anesthesia in women undergoing breast surgery. The primary outcome was the quality of the emergence after general anesthesia (bucking and coughing). The secondary outcomes were PONV and postoperative pain.

## Participants and methods

All procedures were performed at Zadar General Hospital, a single-center regional medical hospital, between July 2007 and December 2013. This study was retroactively registered in ISRCTN because at the time when it was approved by an institutional review board and recruitment started, registration was not required. Written informed consent was obtained from all patients before any study procedures were performed. This prospective, double-blinded, randomized controlled trial was approved by the Institutional Ethics Committee, General Hospital Zadar (01-1280/07). The study was conducted in accordance with the principles of the Declaration of Helsinki.

### Participants

We enrolled 67 adult women with American Society of Anesthesiologists physical status I to II undergoing breast tumor surgery (lumpectomy, simple mastectomy, radical or modified radical mastectomy). Exclusion criteria were expected difficult intubation, multiple intubation attempts, the history of sore throat, cough and respiratory infections, or having been intubated within the last two months, chronic obstructive lung disease, asthma, treatment with β-blocking agents, known hypersensitivity to drugs used in the study protocol, and obesity (body mass index >30 kg/m^2^). Predictors for PONV were also assessed. Patients were not included if the protocol was broken or conditions arose that influenced outcomes during the surgery, such as unexpected intraoperative drug allergy, severe intraoperative hypotension lasting more than three minutes, perioperative hypoxia lasting more than one minute, excessive blood loss, difficult intubation, or serious postoperative surgical complications.

### Management of anesthesia

All patients received 7.5 mg of midazolam *per os* (PO) 1 hour before the surgery with no prophylactic antiemetics, which was the standard of care at the hospital at the time of the study. We used standard monitoring, including electrocardiography, noninvasive blood pressure, pulse oximetry, and capnography. Anesthesia was induced with thiopental 5 mg/kg, fentanyl 1 μg/kg, and vecuronium 0.1 mg/kg. Patients were manually ventilated via face mask with oxygen 6 L/min for three minutes before intubation. An endotracheal tube (ETT) 7.5 mm was used. ETT cuff pressure was measured with an analog manometer to avoid increases higher than 20 cmH_2_O owing to diffusion of nitrous oxide (N_2_O). Anesthesia was maintained with sevoflurane 0.8%-1.5% concentration ~ 1 minimum alveolar concentration (MAC) and 50% N_2_O in oxygen with fresh gas flow 2 L/min. Supplemental bolus doses of fentanyl (1 μg/kg) were added to keep heart rate (HR) and blood pressure within 20% of baseline values but not within the last 30 minutes before extubation. Additional vecuronium was added to maintain one to two twitches on the train-of-four (TOF) monitor. Patients’ lungs were mechanically ventilated to maintain normocapnia (end tidal CO_2_ ~ 35 mm Hg). All patients received about 10 mL/kg/h of crystalloids during surgery.

### Randomization

Patients were randomized by computer-generated random numbers to receive either lidocaine (GLido) or the same volume of saline (GSaline) on the emergence from anesthesia. At the end of skin closure, volatile anesthetic and N_2_O were switched off, neostigmine 2.5 mg and atropine 1 mg for neuromuscular blockade reversal were given, and the fresh gas inflow rate was increased to 7 L/min of 100% oxygen. At that time, patients received 2% lidocaine (1.5 mg/kg) or the same amount of saline. No physical stimulations were applied during emergence. Patients were extubated when they achieved a spontaneous respiratory rate greater than 10 per minute with end-tidal CO_2_≤45 mm Hg and minimum tidal volume of 300 mL, reversal of neuromuscular blockade TOF 4 with sustained tetany, eye opening, strong hand grip, or when demonstrating intentional movement of the extremities, raising the head, or attempting to self-extubate.

### Outcome measures

Early recovery times (from delivery of 100% oxygen to first eye opening and response to verbal commands, to extubation and orientation to time and place) were recorded by a blinded anesthesiologist who did not perform anesthesia. A simple verbal order to “open your eyes,” “squeeze my hand,” “open your mouth,” or “stick out your tongue” was given every 15 seconds during emergence. The number of bucking episodes before extubation and the number of cough and sore throat episodes at one, three, and five minutes after extubation, as well as at two and 24 hours postoperatively were recorded. The quality of tracheal extubation was evaluated on a five-rating scale (1 = no cough or buck; 2 = very smooth, minimal coughing; 3 = moderate coughing; 4 = large degree of coughing or bucking; and 5 = poor extubation, very uncomfortable) (2). Systolic, diastolic, and mean blood pressures, heart rate (HR, beats per minute [bpm]) before induction of anesthesia and immediately before and after extubation were also measured.

An anesthesiologist who was blinded to the study protocol noted complications during emergence in operating room (OR): laryngospasm, bronchospasm, cessation of breathing (number of episodes and duration in seconds), cyanosis, SpO_2_<90%, aspiration of gastric contents and secretions, severe restlessness/agitation (bites ETT, throws self around, kicks hands and feet), vomiting and/or retching, hypertension >35% of preoperative values, tachycardia >35% of preoperative values, bradycardia, or hypotension. The same blinded anesthesiologist estimated the quality of emergence (0 = calm patient, no adverse events, cooperative patient; 1 = calm patient, one adverse event, patient trying to cooperate; 2 = restlessness, one adverse event, uncooperative patient; 3 = restless, at least two adverse events, trying to cooperate; 4 = restless, at least two complications, uncooperative patient).

A modified Aldrete score (0-10 points) was used to determine OR and post-anesthesia care unit discharge readiness (≥9 points). Postoperatively, patients were allowed to drink after three hours, if tolerated. All patients stayed in hospital for at least 24 hours. The incidence of postoperative nausea (PON), vomiting (POV), and the use of rescue antiemetic were assessed at two and 24 hours after surgery. Patients were considered to have had PONV if they experienced at least one episode of nausea, vomiting, or retching, or any combination of these during the initial 24 postoperative hours. POV was defined as at least one episode of vomiting or retching that occurred within 24 postoperative hours. PONV was defined as early (within the first two hours) or late (2-24 postoperative hours). Severity of pain was evaluated with a 100-mm visual analog scale (VAS) at the same time points (0 = no pain to 100 = maximum pain). The same blinded anesthesiologist collected postoperative data. Rescue antiemetic (metoclopramide 0.4 mg/kg IV) was given to patients who experienced two or more episodes of vomiting and/or retching within 30 min, any nausea lasting more than 15 min, or nausea VAS score 50 mm or greater, or when they requested treatment. The pain VAS score and the total amount of postoperative opioids were recorded at two and 24 h after surgery. Diclofenac 75 mg intramuscular injection (IM) was given immediately at the arrival to the recovery room. For severe pain (VAS>40 mm), meperidine up to 100 mg IV was used and repeated four hours later if needed.

### Statistical analysis

The sample size was calculated on the assumption that the incidence of coughing at the end of the anesthesia would be in two-thirds of patients (66%) and that lidocaine would decrease by 50% (to one third of the patients, 33%), thus 33 patients in each group for the primary outcome were needed for a power of 0.8 and alpha level of <0.05. We estimated a 10% drop-out rate. Data are presented as mean (SD) or median (range). The normality of distribution was tested with a D'Agostino-Pearson test. The significance of differences between the categorical variables was assessed with a Fisher exact test or chi square test, and that between continuous variables with a Mann-Whitney test a or *t* test. *P* < 0.05 was considered significant. Data analysis was performed with IBM SPSS Statistics, v. 22.0 (IBM Corp, Armonk, NY, USA).

## Results

**Figure 1:**
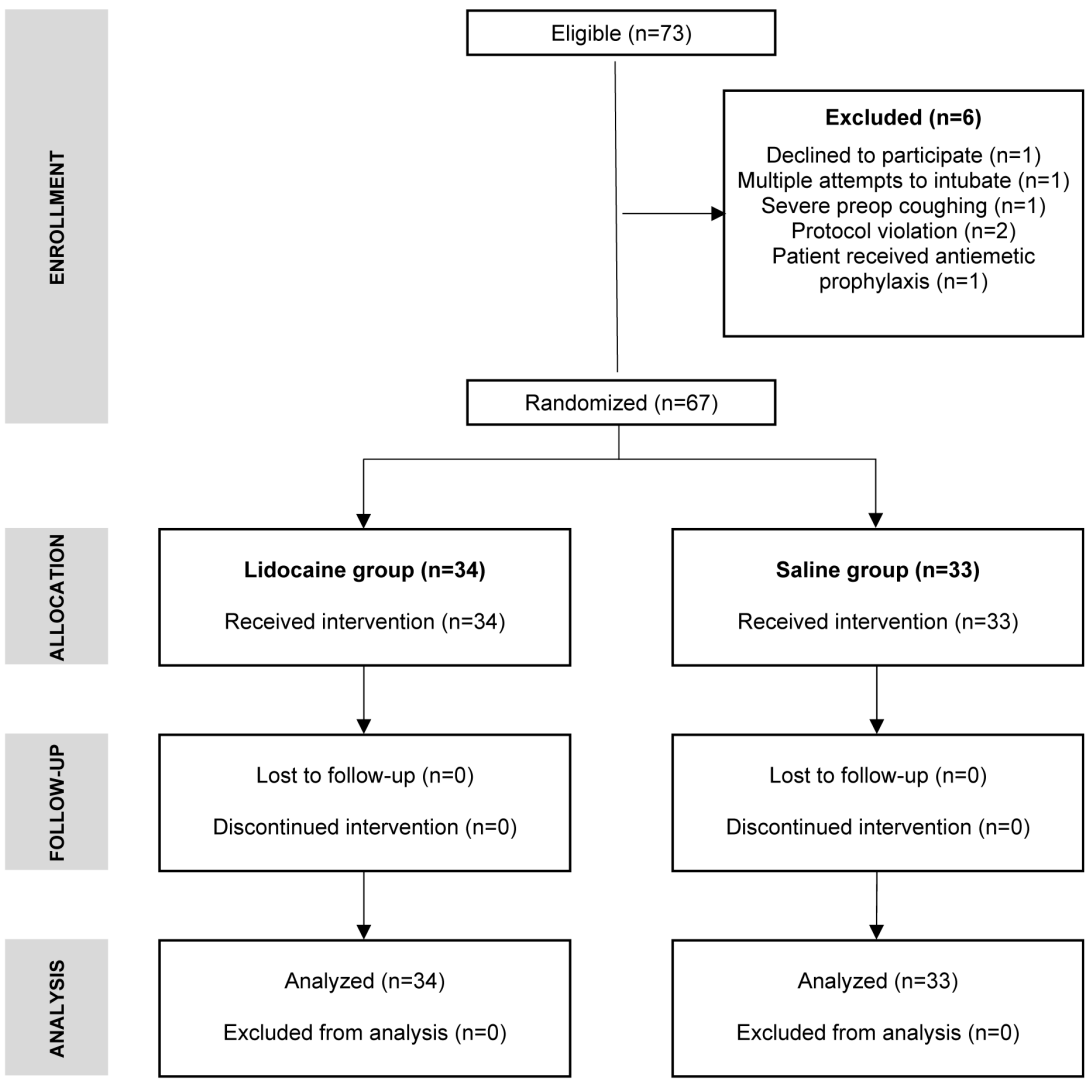
Consolidated Standards of Reporting Trails flow diagram of the study.

Of 73 women who were initially screened for eligibility, six were excluded. Figure 1 presents the flowchart of the study enrollment and the reasons for the exclusion. Of the remaining 67 patients, 34 were randomized into GLido and 33 into GSaline. No significant differences were observed between the groups in demographics, PONV risks (Table 1), and preoperative data (Table 2). Surgery time was significantly shorter (on average 17 minutes) in GLido, but there was no significant difference in anesthesia times even though anesthesia lasted 13 min less in GLido (Table 2).

**Table 1:** Demographic data of patients who received an IV lidocaine 1.5 mg/kg bolus or saline placebo*^†^

Variables	Saline group N = 33	Lidocaine group N = 34
Age (years)	54.2 ± 11.2	49.7 ± 9.4
ASA physical status		
I	22 (66.6)	27 (79.4)
II	11 (33.3)	7 (20.6)
Type of surgery, mastectomy		
with nodes dissection	15 (45.5)	16 (47.0)
simple mastectomy	0	2 (5.8)
lumpectomy	18 (54.5)	16 (47.0)
Weight (kg)	73.6 ± 14.6	67.3 ± 10.9
Height (cm)	167.3 ± 5.9	168.0 ± 5.1
Body mass index (kg/m^2^)	26.2 ± 4.5	23.8 ± 3.6
Apfel score (1-4)	2 (1-4)	2 (1-3)
PONV history	8 (24.2)	8 (23.5)
H/o motion sickness	9 (27.7)	9 (26.4)
Smoking	10 (30.3)	9 (37.5)

*Abbreviations: ASA - American Society of Anesthesiology; H/o - history of; PONV - postoperative nausea and vomiting.

†Data are expressed as mean ± standard deviation, median (min-max value) or n (%).

**Table 2:** Perioperative data of patients who received an IV lidocaine 1.5 mg/kg bolus or saline placebo^†^

Variables	Saline group N = 33	Lidocaine group N = 34	P
Preoperative			
APAIS* score	18.1 ± 6.4	17.3 ± 5.9	
anxiety APAIS score	14 (4-20)	11 (4-20)	
systolic blood pressure (mmHg)	136.4 ± 18.5	128.7 ± 17.2	
diastolic pressure (mmHg)	81.3 ± 8.3	79.3 ± 9.0	
heart rate (beats/min)	74.0 ± 10.3	74.7 ± 13	
Operative			
surgery time (min)	70.8 ± 36.7	53.8 ± 29.8	0.041
anesthesia time (min)	88.7 ± 34.5	75.8 ± 25.4	0.084
fentanyl (μg)	200 (100-350)	200 (100-300)	0.478
thiopental (mg)	350 (250-500)	300 (250-400)	0.051

*APAIS - Amsterdam Preoperative Anxiety and Information Scale (score >11 identifies an anxious patient).

†Data are expressed as mean ± standard deviation or median (min-max value).

A IV lidocaine bolus on emergence was administered about eight minutes before extubation in both groups and did not delay extubation. All emergence variables, either by subjective (emergence and extubation scores) or objective criteria (bucking and hemodynamics), were significantly improved (*P* < 0.001) (Table 3). The greatest improvement was observed in the number of patients who bucked before extubation (91% vs 38%) in GSaline vs GLido, respectively. Furthermore, in GSaline 10 patients had 10 or more episodes of bucking (up to 19 episodes), while only one patient in GLido had more than five episodes (only seven episodes). The average number of bucking episodes was clinically and statistically significantly lower in GLido compared with GSaline (0.97 ± 1.64 vs 5.89 ± 4.99; median 0 [0-7] vs 5 [0-19], *P* < 0.001). The Extubation Quality Score was on average clinically and statistically significantly higher in GLido (1.58 ± 0.66 vs 3.15 ± 1.00; median (1.5 [1-3] vs 3 [1-5], *P* < 0.001), as well as the Quality of Emergence Score (0.35 ± 0.69 vs 1.78 ± 1.20; median 0 [0-3] vs 1 [0-4], *P* < 0.001) (Table 3).

**Table 3:** Emergence data of patients who received an IV lidocaine 1.5 mg/kg bolus or saline placebo*^†^

Variables	Saline group N = 33	Lidocaine group N = 34	P
Eyes opening (sec)	471.5 ± 261.1	487.9 ± 150.2	0.262
Clenching the fist (sec)	510.1 ± 227.4	532.1 ± 265.7	0.679
Extubation (sec)	502.6 ± 245.1	500.9 ± 150.5	0.377
Following commands (sec)	651.0 ± 258.2	653.7 ± 443.7	0.594
Bucking until extubation	30 (90.9)	13 (38.3)	<0.001
Extubation Quality Score^‡^	3 (1-5)	1.5 (1-3)	<0.001
Quality of emergence score			
0	3 (9.0)	25 (73.5)	
1	15 (45.4)	7 (20.6)	
2	6 (18.1)	1 (2.9)	
3	5 (15.1)	1 (2.9)	
4	4 (12.1)	0	<0.001
Before extubation			
SBP (mmHg)	141.4 ± 22.1	132.5 ± 20.2	0.094
MBP (mmHg)	107.4 ± 14.0	101.5 ± 15.3	0.104
DBP (mmHg)	89.8 ± 12.4	86.7 ± 13.0	0.313
HR (beats/min)	83.6 ± 17.8	78.3 ± 16.9	0.219
Post extubation			
SBP (mmHg)	151.7 ± 18.7	137.4 ± 18.7	0.003
MBP (mmHg)	113.4 ± 14.5	105.8 ± 14.4	0.036
DBP (mmHg)	91.5 ± 13.1	89.4 ± 14.0	0.517
HR (beats/min)	87.1 ± 16.8	80.5 ± 15.0	0.098
Changes in hemodynamics			
SBP delta (mmHg)	12 (2-45)	5 (0-18)	<0.001
MBP delta (mmHg)	9 (0-47)	4 (0-17)	0.006
DBP delta (mmHg)	11 (0-51)	3 (0-20)	0.003
HR delta (beats/min)	13 (0-55)	3 (1-26)	<0.001

*Abbreviations: SBP - systolic blood pressure; MBP - mean blood pressure; DBP - diastolic pressure, HR - heart rate.

†Data are expressed as mean±SD, median (min-max value), or n (%).

‡Extubation Quality Score: 1 = no cough or bucking, 2 = very smooth, minimal coughing, 3 = moderate coughing, 4 = large degree of coughing or bucking, 5 = poor extubation, very uncomfortable. Quality of awakening score: 0 = calm patient, no adverse events, cooperative patient; 1 = calm patient, one adverse event, patient trying to cooperate; 2 = restless, one adverse event, uncooperative patient; 3 = restless, at least two adverse events, trying to cooperate; 4 = restless, at least two adverse events, uncooperative patient.

Lidocaine also significantly improved recovery after extubation (Table 4). Post-extubation coughing within the first minute was significantly less frequent in GLido vs GSaline (18% vs 42%; *P* = 0.026). Moreover, only one patient in GLido had more than one coughing episode (three episodes) compared with 10 patients in GSaline (two had 11 and 12 episodes). The average number of coughing episodes was significantly lower in GLido (1.33 ± 0.82 vs 3.93 ± 3.54; median 1 [1-3] vs 3 [1-12]). Beyond the first minute, there was no difference in early coughing between groups, but there was a difference between two and 24 hours (Table 4). Lidocaine reduced the incidence of sore throat tremendously; only two patients reported sore throat in the first 24 h vs 10 patients in GSaline. Also, the total number of patients with complications on emergence was reduced in GLido (18% vs 55%; *P* = 0.002) (Table 4). All patients in GLido had an Aldrete score of 10, while four patients in GSaline had a score of 9 on exit from the OR. The difference was statistically (*P* = 0.039) but not clinically significant. The hemodynamic response on emergence and extubation was significantly attenuated in the GLido group (Table 3). An increase in HR in GLido was only 3 bpm, while in GSaline it was 13, *P* < 0.001.

**Table 4:** Recovery data of patients who received an IV lidocaine 1.5 mg/kg bolus or saline placebo*

Variables	Saline group N = 33	Lidocaine group N = 34	P
Coughing			
0-1 min	14 (42.4)	6 (17.6)	0.026
1-3 min	7 (21.2)	3 (8.8)	0.155
3-5 min	4 (12.1)	6 (17.6)	0.526
2 h	6 (18.2)	2 (0.6)	0.121
2-24 h	9 (27.3)	3 (8.8)	0.049
Sore throat			
2 h	10 (30.3)	1 (2.9)	0.007
2-24 h	10 (30.3)	1 (2.9)	0.007
24 h	16 (48.4)	2 (5.8)	<0.001
All complications	18 (54.5)	6 (17.6)	0.002
Aldrete score	10 (9-10)	10 (10-10)	0.039

*Data are expressed as n (%) or median (minimal-maximal value).

IV lidocaine bolus on emergence reduced PONV at all measurements, but a significant difference was observed only in late PONV (3% vs 24%, *P* = 0.13) and late PON (3% vs 21%, *P* = 0.27) (Table 5). All patients in both groups received diclofenac IM at the end of the surgery. There were no differences in postoperative pain or pain medications between the groups (Table 6). No signs of lidocaine adverse effects or toxicity were noted in any patient.

**Table 5:** Postoperative nausea and vomiting (PONV) data of patients who received an IV lidocaine 1.5 mg/kg bolus or saline placebo*

Variables	Saline group N = 33	Lidocaine group N = 34	P
PONV	13 (39.3)	7 (20.6)	0.157
early (2 h)	11 (33.3)	7 (20.6)	0.368
late (2-24 h)	8 (24.2)	1 (2.9)	0.013
POV	8 (24.2)	3 (8.8)	0.170
early (2 h)	7 (21.2)	1 (3.0)	0.054
late (2-24 h)	6 (18.1)	1 (2.9)	0.054
PON	13 (40.6)	7 (20.6)	0.133
early (2 h)	11 (33.3)	6 (18.1)	0.260
late (2-24 h)	7 (21.2)	1 (2.9)	0.027
Rescue antiemetic	10 (30.3)	3 (8.8)	0.050

*Data are expressed as n (%).

**Table 6:** Postoperative pain of patients who received an IV lidocaine 1.5 mg/kg bolus or saline placebo*

Variables	Saline group N = 33	Lidocaine group N = 34	P
Visual analog scale pain at 2 h	20 (0-70)	22.5 (0-70)	0.432
Visual analog scale pain at 24 h	10 (0-30)	0 (0-30)	0.743
Pethidine (rescue)	11 (34.4)	9 (26.4)	0.667
Diclofenac (second dose)	5 (15.1)	1 (2.9)	0.105
Any rescue	13 (39.4)	10 (29.4)	0.547

*Data are expressed as n (%) or median (minimum-maximum value).

## Discussion

The results of our Lidocaine at the End (LATE) study showed profound effects of IV lidocaine on early and late recovery without delaying extubation when given at the end of the surgery after the skin closure was completed, neuromuscular blockade reversal was given, and anesthetic gases were turned off.

The most striking improvement was the decreased number of patients who bucked before extubation by more than half (GSaline 91% vs GLido 38%), number needed to treat (NNT) of two, as well as the number of bucking episodes (GLido 0 [0-7] vs GSaline 5 [0-19]). Early coughing was also improved (GSaline 42% vs GLido 18%), with a NNT of four. This agrees with the findings of a recent meta-analysis, which showed a NNT of five for postoperative cough (1). To our knowledge, this is the first study to separate bucking before extubation and coughing after extubation, as well as to frequently measure coughing after extubation. We believe that it is important to report bucking before extubation separately, not only for research purposes but also from a clinical standpoint. For instance, anesthesia personnel could respond to patients having episodes of multiple/severe bucking by extubating them before extubation criteria are reached, thus putting them at risk of respiratory complications. Reducing bucking by more than half could significantly improve outcomes, especially in patients who would be completely awake before extubation (difficult intubation or with sleep apnea) after surgery, which demands avoiding bucking. Indeed, in our study, all combined emergence complications were decreased 3-fold (GSaline 55% vs GLido 18%), with NNT 2. Moreover, the quality of emergence measured by a blinded anesthesiologist was smoother in patients who received IV lidocaine, a finding that could be important in high-risk patients.

Although the exact mechanism of how IV lidocaine reduces bucking and coughing is not well understood, the timing of its administration is important. If IV lidocaine is given too early, its effect could already be dissipating. Indeed, IV lidocaine did not reduce post-extubation cough when it was given on average 21 minutes before extubation (3). Even 10 minutes before extubation might be too early (4,5). On the contrary, if it is given too close to extubation it might not work either. For example, when IV lidocaine was given just three minutes before extubation, it had no effect on coughing (6). Between five (7) and eight minutes (our LATE study) before extubation could be a sweet spot for reducing bucking and coughing on emergence. In our study, lidocaine was given when the surgery was completed, gasses were turned off (sevoflurane/50%N_2_O), and reversal was administered, which made extubation time ( ~ 8 minutes) highly predictable. This timing might correlate with IV lidocaine peak plasma concentration after a single IV bolus dose with a rapid decline after five minutes (8).

Our study confirmed the beneficial effect of IV lidocaine on postoperative sore throat. It almost completely eliminated it, with only two patients reporting sore throat within 24 h, with a NNT of 2.4. The meta-analysis (1) showed a NNT of four. The difference could be explained by the timing of IV lidocaine administration because the meta-analysis analyzed all perioperative administrations of IV lidocaine including preinduction. Also, as in most previous studies, IV lidocaine’s influence on hemodynamics was confirmed. Changes in blood pressures were on average 10 mm Hg lower in GLido than in GSaline after extubation. This finding might not be clinically significant, but HR was also lower by 10 bpm, which could be clinically significant. HR in GLido was practically unchanged with a delta of only 3 bpm.

The influence of IV lidocaine bolus administered on emergence on PONV was less investigated, and the evidence was of very low quality. A meta-analysis of perioperative infusion of lidocaine showed a reduction in PONV (9). The incidence of early PONV was 20.1% (45:218) of participants in the lidocaine group and 28.4% (63:222) of participants in the control group, with a NNT of 12. Late PONV (within 72 h postoperatively) occurred in 26.6% (154: 545) of lidocaine participants and in 35.6% (192:539) of control participants, with a NNT of 11. A meta-analysis of IV bolus of lidocaine in pediatric patients showed that intravenous lidocaine may reduce the incidence of PONV, but the quality of the evidence was very low (10). The incidence of PONV within 24 hours after anesthesia was 3.73% in the lidocaine group and 4.87% in the control group (NNT of 100) (10). In our study, lidocaine reduced the incidence of PONV within the first 24 h almost by half (GSaline 39.3% vs GLido 20.6%, NNT of 5.5), but the difference was not significant, probably because the study was underpowered. All other incidences were reduced in both early and late POV/PON but only late PONV reached significance. IV lidocaine bolus also borderline non-significantly reduced the use of rescue antiemetics (GSaline 30.2% vs GLido 8.8%, NNT of 4.5). This suggests that IV lidocaine may clinically significantly reduce PONV and the use of antiemetics. One of the differences between studies is the type of surgery and the use of PONV prophylaxis. In our study, we did not use any PONV prophylaxis, which was the standard in our hospital at the time of the study. This gave us a “raw” effect of IV lidocaine bolus, but its clinical benefit may be attenuated by routine use of antiemetics.

Limitations of our study include that it was done in one center, in only one type of surgery, and during relatively short duration of surgery. Our results may not be generalized to other types of surgery. Since we administered IV lidocaine at the end of the surgery, one may expect similar results in longer surgeries. Another limitation is the small number of patients in each group, which may render the study underpowered for the secondary outcome (PONV), as discussed above. The strength of our study is that anesthesia emergence was standardized and extubation timing was highly predictable. We believe that it is important to separate extubation bucking from postoperative coughing. These conditions may have different etiologies: pre-extubation bucking is mostly, if not exclusively, caused by direct ETT stimulation of the trachea but post-extubation coughing is mostly caused by secretions and injury of the tracheal mucosa. We think that in future studies pre-extubation bucking should be separately reported from post-extubation/post-operative coughing. An additional strength is that our study showed “raw” (without any PONV prophylaxis) effect of IV lidocaine on PONV.

In summary, our LATE study showed an overwhelming beneficial effect of an IV lidocaine 1.5 mg/kg bolus given eight minutes before extubation on bucking, coughing, sore throat, increase in HR and BP, as well as PONV without any delay in extubation time.
